# Influence of Hypertension on Longitudinal Changes in Brain Glucose Metabolism Was Modified by the APOE4 Allele Among Cognitively Normal Older Individuals

**DOI:** 10.3389/fnagi.2020.00085

**Published:** 2020-04-02

**Authors:** Rui Zhou, Hao Chen, Fanhao Ye, Shiwei Huang, Jie Zhang

**Affiliations:** ^1^Department of Cardiology, Wenzhou People’s Hospital, Wenzhou, China; ^2^Independent Researcher, Hangzhou, China

**Keywords:** brain glucose metabolism, FDG-PET, hypertension, APOE4, longitudinal study

## Abstract

**Objective:**

To examine whether the influence of hypertension (HTN) status on longitudinal changes in brain glucose metabolism was modified by the apolipoprotein 4 (APOE4) status among older people with normal cognition.

**Methods:**

In this study, we included 217 older individuals with normal cognition from the Alzheimer’s Disease Neuroimaging Initiative (ADNI) study. Participants were divided into the HTN and no HTN groups based on self-reported medical history. Brain glucose metabolism was assessed by 18F-fluorodeoxyglucose-positron emission tomography (FDG-PET). Linear mixed model was fitted to examine the association between the HTN × APOE4 interaction and longitudinal changes in brain glucose metabolism after controlling for several covariates.

**Results:**

In the present study, we found that the association between HTN status and longitudinal changes in brain glucose metabolism varied as a function of the APOE4 status, such that the HTN/APOE4+ group showed a steeper decline in FDG SUVR than all other groups (No HTN/APOE4-, HTN/APOE4-, and No HTN/APOE4+). Nevertheless, there was no significant difference in the rate of decline in FDG SUVR among other groups (No HTN/APOE4-, HTN/APOE4-, and No HTN/APOE4+).

**Conclusion:**

The APOE4 genotype interacted with hypertension status to affect longitudinal changes in brain glucose metabolism among older individual with normal cognition, such that the HTN/APOE4+ group showed a steeper decline in FDG SUVR than other groups.

## Introduction

Deteriorating brain glucose metabolism is a key feature of Alzheimer’s disease (AD) and precedes the clinical onset of AD (Small et al., 1995; Reiman et al., 1996; Li et al., 2008; Chen and Zhong, 2013). Cerebral glucose metabolic rates, assessed by 18F-fluorodeoxyglucose-positron emission tomography (FDG-PET), provide an crucial measure of the dysfunction of neurons and synapses in living human (De Leon et al., 2001; Dubois et al., 2007).

The apolipoprotein E (APOE) gene is the most important genetic risk factor for late-onset sporadic AD (Safieh et al., 2019). This gene has three polymorphic forms, ε2, ε3, ε4; and the APOE4 allele increases the risk of cognitive decline and AD dementia (Safieh et al., 2019). However, most, but not all FDG studies have suggested that the APOE4 allele is associated with reduced levels of brain glucose metabolism (Reiman et al., 1996, 2004, 2005; Corder et al., 1997; Samuraki et al., 2012). There is a possibility that the APOE4 allele may interact with other cardiovascular diseases [e.g., hypertension (HTN)] to affect the levels of brain glucose metabolism. In line with this notion, previous observational studies indicated that APOE4 and HTN act synergistically to influence cognitive performance, subcortical white matter integrity, and cortical amyloid accumulation (Peila et al., 2001; De Leeuw et al., 2004; De Frias et al., 2014; Oberlin et al., 2015; Jeon et al., 2019). However, no prior studies have attempted to assess the contributions of the APOE4 and HTN status to longitudinal changes in brain glucose metabolism among older individuals with normal cognition.

In this study, among older individuals with normal cognition, we hypothesized that the interaction between APOE4 and HTN is associated with longitudinal changes in brain glucose metabolism, such that APOE4 carriers with a history of hypertension (APOE4 + /HTN) show a steeper rate of decline in brain glucose metabolism than other groups (APOE4-/HTN, APOE4-/No HTN, and APOE4/No HTN).

## Materials and Methods

### Alzheimer’s Disease Neuroimaging Initiative (ADNI)

Longitudinal data used in the preparation of this work were extracted from the Alzheimer’s Disease Neuroimaging Initiative (ADNI) database^1^. The ADNI study was conducted with the primary aim of discovering potential biomarkers of cognitive decline for clinical trials. At ADNI centers, local institutional review boards approved the study, and each participant provided written informed consent.

### Participants

At baseline, we included a total of 217 older individuals with normal cognition. In the present analysis, we included subjects who had baseline and follow-up measurement of brain glucose metabolism. The sample size at baseline and each follow-up visit were displayed in Table 1. Participants with normal cognition had a Clinical Dementia Rating (CDR) (Morris, 1993) of 0 and a mini-mental state examination (MMSE) (Folstein et al., 1975) of 24 or higher.

**TABLE 1:** Demographic and clinical variables by HTN status.

**Variables**	**No HTN (*n* = 121)**	**HTN (*n* = 96)**	***P* values**
Age, years	74.2 ± 5.99	75.2 ± 5.69	0.2
Education, years	16.4 ± 2.65	16.4 ± 3.03	0.8
Female gender, n (%)	54 (44.6)	39 (40.6)	0.55
APOE4, n (%)	30 (24.8)	31 (32.3)	0.22
MMSE scores	29.1 ± 1.13	29 ± 1.26	0.48
FDG SUVR	1.31 ± 0.11	1.29 ± 0.11	0.18
Serum glucose, mg/dL	97.7 ± 18.3	102 ± 22.9	0.16
Total cholesterol, mg/dL	191 ± 39.6	185 ± 37.5	0.27
Triglyceride, mg/dL	136 ± 86.2	147 ± 89	0.35
**Numbers of participants at baseline and each follow-up visit, n**			
Baseline	121	96	
1 year	41	42	
2 years	112	91	
3 years	36	34	
4 years	29	25	
5 years	18	18	
6 years	24	17	
7 years	14	16	
8 years	3	2	
11 years	4	4	
12 years	1	0	

*HTN, hypertension; MMSE, mini-metal state examination; FDG SUVR, fluorodeoxyglucose standardized uptake value ratios.*

### Hypertension (HTN) Status

Participants were further categorized into the No HTN (*n* = 121) and HTN (*n* = 96) groups according to the self-reported history of HTN. Several search terms (hypertension, high blood pressure, and HTN) were used to screen medical history of ADNI’ subjects.

### APOE Genotyping

APOE4 genotypes of ADNI’s participants were extracted from the ADNI website^2^. Participants were divided into the APOE4- (absence of the APOE4 allele) and APOE4 + (presence of at least one APOE4 allele) groups.

### Measurement of Brain Glucose Metabolism

Cerebral metabolic rates for glucose were examined using FDG-PET by Susan Landau and William Jagust’s group, Helen Wills Neuroscience Institute, UC Berkeley and Lawrence Berkeley National Laboratory. The neuroimaging techniques have been described previously (Landau et al., 2011). Five pre-defined regions of interest (MetaROIs) were identified according to coordinates reported frequently in previously FDG investigations comparing healthy controls, MCI, and AD patients. These five crucial hypometabolic ROIs included left angular gyrus, right angular gyrus, left inferior temporal gyrus, right inferior temporal gyrus, and bilateral posterior cingulate gyrus. In the present analysis, FDG standardized uptake value ratios (SUVR) were defined by averaging FDG uptake of these five regions and then dividing by FDG uptake of pons and cerebellum (a reference region) (Jagust et al., 2010; Landau et al., 2011). The reference region (the cerebellum and pons) was used in order to reduce between-subject nuisance variability in trace uptake.

### Statistical Analysis

Group differences were examined with ANOVA tests for continuous parameters and Chi-squared tests for categorical parameters. In an effort to assess the association of the HTN^∗^APOE4 interaction with longitudinal changes in FDG SUVR among cognitively normal older individuals, we performed the linear mixed model including the three-way HTN × APOE4 × Time interaction term. This model also included main effects of age, educational years, gender, serum glucose, triglyceride, total cholesterol and their interactions with time, along with a random intercept for each subject. Finally, to examine interactions between HTN and APOE4 genotype, longitudinal changes in FDG SUVR across all pairwise group contrasts (No HTN/APOE4-, HTN/APOE4-, No HTN/APOE4+, and HTN/APOE4+) were conducted. The Tukey method was used for multiple comparisons correction. All statistical work was conducted using R version 3.6.0.

## Results

### Demographic and Clinical Variables by HTN Status

At baseline, a total of 217 older individuals with normal cognition was included. As shown in Table 1, no significant differences in demographics (age, education, gender and APOE4 genotype) and clinical variables (MMSE scores, FDG SUVR, serum glucose, total cholesterol, and triglyceride) were found between two groups (No HTN vs. HTN).

### Demographics by HTN and APOE4 Status

Our participants were divided into four groups according to HTN and APOE4 status (Table 2). Demographics were compared between these four groups. However, there were no significant differences in age, educational years or the percentage of female gender across the four groups (all *p* > 0.05).

**TABLE 2:** Cognitively normal older adults by HTN and APOE4 status.

**Variables**	**No HTN/APOE4−**	**HTN/APOE4−**	**No HTN/APOE4+**	**HTN/APOE4+**
N	91	65	30	31
Age, years	74.4 ± 5.72	76 ± 5.29	73.4 ± 6.81	73.5 ± 6.19
Education, years	16.4 ± 2.67	16.6 ± 3.2	16.5 ± 2.66	15.8 ± 2.6
Female, n (%)	40 (44)	26 (40)	14 (46.7)	13 (41.9)

*HTN, hypertension.*

### Longitudinal Change Models

**FIGURE 1:**
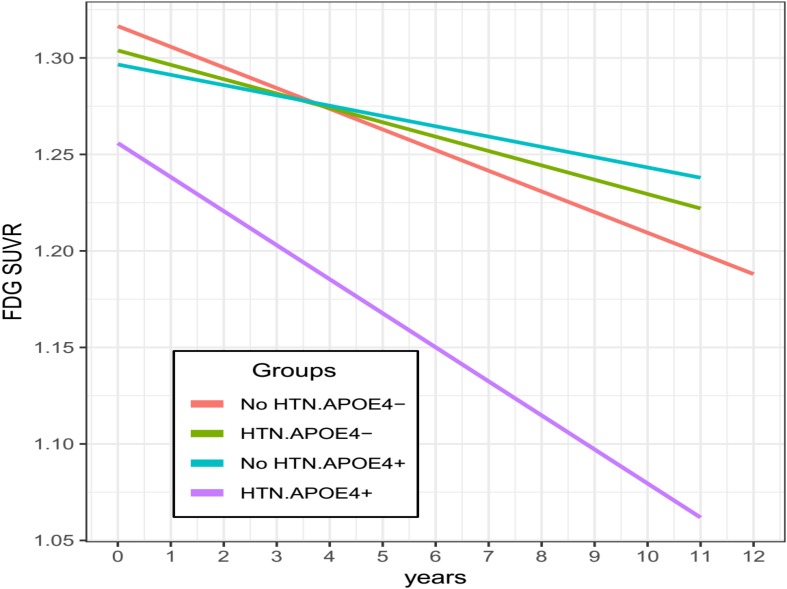
Longitudinal changes in FDG SUVR stratified by HTN and APOE4 status. Compared with other groups, the HTN/APOE4 + group demonstrated significantly steeper decline in FDG SUVR. However, there was no significant difference in the rate of decline in FDG SUVR among other groups. HTN, hypertension.

To assess the contributions of HTN and APOE4 status to longitudinal changes in FDG SUVR, the linear mixed model was fitted. As shown in Table 3, we found that the 3-way interaction between HTN, APOE4, and time was significant for FDG SUVR (estimate = −0.0181, SE = 0.0054, *p* = 0.0008). To better understand this interaction, longitudinal changes in FDG SUVR across all pairwise group contrasts (No HTN/APOE4-, HTN/APOE4-, No HTN/APOE4+, and HTN/APOE4+) were conducted (Figure 1 and Table 4). Compared with other groups, the HTN/APOE4+ group demonstrated significantly or marginally significant steeper decline in FDG SUVR (Figure 1 and Table 4). However, there was no significant difference in the rate of decline in FDG SUVR among other groups (No HTN/APOE4-, HTN/APOE4-, and No HTN/APOE4+; Figure 1 and Table 4).

**TABLE 3:** Linear mixed models examining the influence of the HTN^∗^APOE4 interaction on longitudinal changes in FDG SUVR.

**Predictors**	**Estimate**	**SE**	***P* values**
Age × Time	−0.0004	0.0003	0.1175
Education × Time	−0.0006	0.0004	0.1672
Female gender × Time	−0.0001	0.0028	0.767
Serum glucose × Time	0	0.0001	0.7635
Triglyceride × Time	0	0	0.0608
Total cholesterol × Time	0	0	0.1755
HTN × Time	0.0037	0.0028	0.1898
APOE4 × Time	0.0059	0.0038	0.1262
HTN × APOE4 × Time	−0.0180	0.0054	0.0008

*HTN: hypertension. The model was adjusted for main effects of age, education, gender, serum glucose, triglyceride, and total cholesterol (estimate not displayed). Estimates represent the amount of change in FDG SUVR every year.*

**TABLE 4:** Comparisons across HTN/APOE4 groups.

**Contrast**	**Estimate**	**SE**	***P* value**
No HTN/APOE4- vs. HTN/APOE4-	−0.0037	0.0028	0.5645
No HTN/APOE4- vs. No HTN/APOE4+	−0.0059	0.0039	0.4299
No HTN/APOE4- vs. HTN/APOE4+	0.0085	0.0036	0.0784
HTN/APOE4- vs. No HTN/APOE4+	−0.0022	0.0041	0.9506
HTN/APOE4- vs. HTN/APOE4+	0.0122	0.0037	0.0061
No HTN/APOE4+ vs. HTN/APOE4+	0.0144	0.0047	0.0114

*HTN, hypertension; FDG SUVR, fluorodeoxyglucose standardized uptake value ratios. Estimates represent the amount of change in FDG SUVR every year.*

## Discussion

In this study, we hypothesized that the APOE4 allele would moderate the influence of HTN status on longitudinal changes in brain glucose metabolism among cognitively normal older people. In line with this hypothesis, we observed that the relationship between HTN status and longitudinal changes in brain glucose metabolism varied as a function of the APOE4 status, such that the HTN/APOE4+ group showed a steeper decline in FDG SUVR than all other groups (No HTN/APOE4-, HTN/APOE4-, and No HTN/APOE4+). Nevertheless, there was no significant difference in the rate of decline in FDG SUVR among other groups (No HTN/APOE4-, HTN/APOE4-, and No HTN/APOE4+).

The finding that the HTN/APOE4+ group had a steeper decline in FDG SUVR than all other groups among cognitively normal older people is novel. Consistent with our finding, previous observational studies showed that the influence of hypertension on cognitive deficits, medial temporal atrophy, subcortical white matter lesions, cortical amyloid deposition, and tau phosphorylation was greater in APOE4 carriers than in APOE4 non-carriers (Peila et al., 2001; De Leeuw et al., 2004; Korf et al., 2004; Den Heijer et al., 2005; Kester et al., 2010; De Frias et al., 2014; Andrews et al., 2015; Oberlin et al., 2015; Jeon et al., 2019). Collectively these data and ours indicate that the impact of hypertension on neuronal damage and synapse loss appears to be exacerbated by the APOE4 allele.

The mechanisms by which the APOE4 allele could modify the association of hypertension on longitudinal changes in FDG SUVR are not very clear. However, there are several potential possibilities for the APOE4 × Hypertension interaction in relation to longitudinal changes in FDG SUVR. First, APOE is thought to play an important role in the response to neuronal damage by redistributing lipids to facilitate the regeneration of neuronal axons and maintaining the structure and function of the microtubules (Handelmann et al., 1992). Compared with wild-type mice, APOE-deficient mice have demonstrated much greater ischemic neuronal injury after ischemic episodes (Horsburgh et al., 1999). However, this beneficial effect is dependent on polymorphic forms: the APOE3 allele appears to facilitate the repair process, while the APOE4 allele tends to retard the process (Nathan et al., 1994; Bellosta et al., 1995; Teter et al., 1999). Therefore, it is likely that the influence of hypertension on neuronal structure would be greater in APOE4 carriers because of their limited ability to promote the repair process.

Second, it has been reported that a history of hypertension is associated with higher levels of neuritic plaques and neurofibrillary tangles (Sparks et al., 1995; Petrovitch et al., 2000). Similarly, compared to APOE4 non-carriers, APOE4 carriers shows a greater amount of amyloid and tau pathologies (Leoni, 2011). More importantly, a recent study using [11C]-Pittsburgh-compound-B-positron emission tomography showed that in APOE4 carriers, hypertension was associated with increased cortical Aβ accumulation (Jeon et al., 2019), which could contribute to brain glucose hypometabolism (Lowe et al., 2014). Therefore, the effect of hypertension on neuronal injury, measured by FDG SUVR, would be expected to be larger for APOE4 carriers.

Several limitations should be noted. First, participants in the ADNI study were highly educated and had fewer comorbidities. For instance, participants who had the Hachinski Ischemic Scale (HIS) score of 5 or higher were excluded from the ADNI study. Thus, this may limit the generalizability of our findings. Further studies, especially population-based studies, were needed to replicate our results. Second, the present study primarily focused on the association of the APOE4^∗^hypertension interaction with changes in FDG SUVR. It would be interesting to examine the association of this interaction with clinical progression and other AD-related markers, including cognitive function and CSF AD pathologies.

## Conclusion

In conclusion, the APOE4 interacted with hypertension status to affect longitudinal changes in brain glucose metabolism among older individual with normal cognition, such that the HTN/APOE4+ group showed a steeper decline in FDG SUVR than other groups (Nos HTN/APOE4-, HTN/APOE4-, and No HTN/APOE4+).

## Data Availability Statement

The datasets generated for this study will not be made publicly available. Datasets can be found at the ANDI website (http://adni.loni.usc.edu).

## Ethics Statement

The studies involving human participants were reviewed and approved by the ADNI centers approved the ADNI study. The patients/participants provided their written informed consent to participate in this study.

## Author Contributions

JZ and SH conceived and designed the study. RZ, HC, and FY performed the research and analyzed the data. RZ wrote the manuscript. All authors approved the final version of the manuscript.

## Conflict of Interest

The authors declare that the research was conducted in the absence of any commercial or financial relationships that could be construed as a potential conflict of interest.

## Footnotes

1 www.adni-info.org

2 adni.loni.usc.edu
